# Molar root canal treatment performed by undergraduate dental students; an observational study of procedural errors and student perception

**DOI:** 10.1186/s12909-024-05397-z

**Published:** 2024-04-22

**Authors:** Ahmad M. El-Ma’aita, Sari A. Mahasneh, Maryam A. Hamandi, Mohammad A. Al-Rabab’ah

**Affiliations:** 1 Restorative Dentistry Department, University of Jordan, Amman, Jordan; 2 Jordan University Hospital, Amman, Jordan

**Keywords:** Dental education, Manual instrumentation, Procedural errors, Root canal treatment, Rotary instrumentation

## Abstract

**Background:**

Molar root canal treatment (RCT) is challenging and requires training and specific skills. Rotary instrumentation (RI) reduces the time needed for instrumentation but may increase the risk of certain procedural errors. The aims of this study were to evaluate the quality of molar RCTs provided by undergraduate students, to compare the prevalence of procedural errors following manual and RI, and to assess the students’ self-perceived confidence to perform molar RCT without supervision and their preference for either manual or RI.

**Methods:**

Molar RCTs performed by the final year students were evaluated radiographically according to predefined criteria (Appendix 1). The procedural errors, treatment details, and the students’ self-perceived confidence to perform molar RCT and their preference for either manual or RI were recorded. Descriptive statistics were performed, and the Chi-squared test was used to detect any statistically significant differences.

**Results:**

60.4% of RCTs were insufficient. RI resulted in more sufficient treatments compared with MI (49% vs. 30.3% respectively. X^2^: 7.39, *p* = 0.007), required fewer visits to complete (2.9 vs. 4.6 respectively. X^2^: 67.23, *p* < 0.001) and was the preferred technique by 93.1% of students. The most common procedural errors were underextension of the root canal obturation (48.4%), insufficient obturation (45.5%), and improper coronal seal (35.2%) without a significant difference between the two techniques. 26.4% of the participating students reported that they did not feel confident to perform molar RCT without supervision.

**Conclusion:**

The quality of molar RCT provided by UG students was generally insufficient. RI partially improved the technical quality of RCT compared with MI. UG students need further endodontic training and experience before they can safely and confidently practise molar RCT.

**Supplementary Information:**

The online version contains supplementary material available at 10.1186/s12909-024-05397-z.

## Background

Root canal treatment (RCT) aims to control intra-radicular infection and prevent or treat apical periodontitis [1]. It comprises chemo-mechanical disinfection, where canals are instrumented and shaped to their full extension and disinfected using irrigants and medicaments, and then obturated in three-dimension to ensure a fluid-tight seal apically, laterally and coronally [2, 3]. RCT is a technically demanding procedure that requires specific training and skills especially in molar teeth. Undergraduate (UG) students may lack the skills and experience necessary to carry out molar RCT. Only 13–47% of RCTs completed by UG students were of acceptable quality [4–8]. 

Procedural errors may occur during RCT even with experienced clinicians. They can manifest as errors in length control, instrumentation-related errors and insufficient obturation. The presence of such errors can jeopardize canal debridement, which may, in turn, compromise the outcome of endodontic treatment [9, 10]. Rotary instrumentation (RI) uses motor driven nickel-titanium (NiTi) instruments that are super-elastic and demonstrate higher resistance to torsional failure compared with stainless steel instruments [11, 12]. RI was shown to perform better than manual instrumentation (MI) when used by unexperienced clinicians and resulted in fewer procedural errors [13, 14]. Its integration into UG dental education was suggested [13]. 

Research that evaluates student learning outcome should utilize competency measures as the main outcome [15]. However, students’ self-perceived confidence can influence the student’s ability to demonstrate competency and may be useful as a secondary outcome [16]. The students’ self-perceived confidence in performing endodontic procedures was the lowest for performing RCT of maxillary and mandibular molars among a list that involves a wide range of endodontic procedures [17]. Less than 40% of new dental graduates were confident to perform endodontic treatment on multirooted teeth [18]. 

The need for this study stems from multiple observations by our faculty supervising UG endodontic work that students struggled to perform molar RCT and that procedural errors were frequent. The aims of this observational study were (a) to assess the quality of molar RCTs provided by UG students, (b) to compare the prevalence of procedural errors associated with manual and rotary instrumentation and (c) to assess the students’ self-perceived confidence to perform molar RCT without supervision and their preference for either manual or RI.

## Methods

The protocol of this study was approved by the review board committee of the Jordan University Hospital (ref 10/2022/1726). Informed consent was obtained from the participating students and their patients. All final year dental students at the University of Jordan (*n* = 149) were invited to participate in this study. No exclusion criteria were implemented, and no students form other colleges were included. Molar RCTs performed by the final year dental students at the University of Jordan between October 2021– May 2022 were evaluated radiographically according to predefined criteria (Appendix 1). All the treated cases were of low difficulty according to the American Association of Endodontists’ case difficulty assessment form [19]. All RCTs were performed under rubber dam isolation. Working length was determined using apex locators and confirmed with a working length radiograph. Canal instrumentation was performed either manually using the step back technique, or with rotary instrumentation using the ProTaper Gold® system (Dentsply Maillefer, Baillagues, Switzerland). Obturation was carried out with gutta percha and resin sealer (AH-plus, Dentsply DeTrey, Konstanz, Germany) using cold lateral condensation. Students were supervised by faculty throughout the treatments provided. However, different supervisors were allocated to different groups of students. It was not possible to assign the same supervisors for all students due to the busy clinics schedule.

Treatment details including the number of visits used to complete the treatment, the students’ preference for either instrumentation technique, and their self-perceived confidence to perform RCT without supervision were recorded using an online questionnaire (Appendix 2). The questionnaire was designed was piloted on 10 students and was tested for face and content validity. The first 10 respondents were asked to complete the survey once again after one week to ensure the survey was reliable.

Post-obturation radiographs (manual type E films, Kodak, Carestream Health, Rochester, NY, USA) were assessed in a dark room using an X-ray viewer (Dentsply Rinn, Konstanz, Germany). Procedural errors that were detectable on the post-obturation peri-apical radiograph were recorded, and each completed RCT was marked as either sufficient or insufficient (Appendix 1). The first 10 RCTs were jointly evaluated by 3 assessors (AE, MA, and SM) following a discussion of the errors detected to ensure good calibration of the assessors. The rest of the RCTs were evaluated independently by 2 clinicians (AE and MA). In cases where there was a disagreement, the third experienced consultant endodontist (SM) was consulted. Descriptive statistics as well as the Chi-squared test were used.

## Results

A total of 109 students consented to participate. 104 students performed molar RCTs using both techniques and 5 students only performed manual instrumentation. Post-obturation radiographs of 213 RCTs were assessed (109 using MI and 104 using RI). 75.6% of treated teeth were first molars (26.8% maxillary and 48.8% mandibular) while 24.4% were second molars (10.3% maxillary and 14.1% mandibular). The overall mean number of visits required to complete the treatment was 3.7 (median: 4, standard deviation: 1.55). RI enabled the students to complete their treatment in fewer visits compared with MI (2.9 vs. 4.6 visits respectively, X^2^: 67.23, *p* < 0.001).

**Fig. 1:**
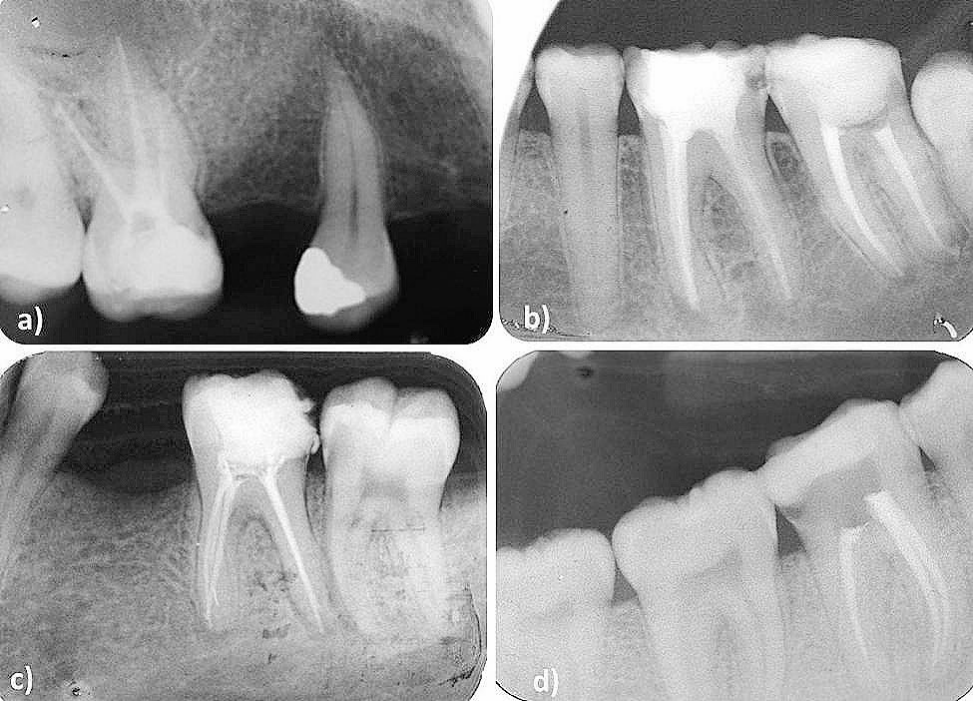
Procedural errors detected in the post-obturation radiographs; (**a**) under-extension of obturation in the mesio-buccal root of the maxillary right first molar, (**b**) canal transportation in the mesial root of the mandibular left first molar, (**c**) separated instrument in the mesio-lingual root of the mandibular left first molar, and (**d**) Defective coronal restoration in the mandibular right first molar

The procedural errors (as defined in Appendix 1) and their prevalence are summarised in Table 1. Examples of the procedural errors are illustrated in Fig. 1.

**Table 1:** The procedural errors detected in the RCTs provided and their prevalence

Procedural error	Prevalence	X^2^-instrumentation technique (*P* value)	X^2^-arch (maxilla vs. mandible) (*P* value)
Improper access cavity	7.5%	–	0.253 (0.615)
Missed canals	5.6%	–	1.564 (0.211)
Under extension	48.4%	1.385 (0.239)	1.855 (0.173)
Over extension	19.2%	4.325 (0.038)*	0.416 (0.519)
Improper apical instrumentation size	7.5%	2.748 (0.097)	1.084 (0.298)
Ledge formation	2.8%	0.003 (0.953)	0.441 (0.507)
Canal transportation	5.2%	3.897 (0.048)*	2.652 (0.103)
Access cavity-related perforation	2.8%	–	1.104 (0.293)
Instrumentation-related perforation	1.4%	3.19 (0.074)	1.794 (0.180)
Strip perforation	0%	–	–
Separated instrument	3.8%	2.279 (0.131)	0.001 (0.980)
Sealer extrusion	3.8%	1.889 (0.169)	0.521 (0.471)
Insufficient obturation	45.5%	5.654 (0.017)	0.742 (0.389)
Improper coronal seal	35.2%	–	0.822 (0.365)

*Denotes a statistically significant difference

The overall technical quality of RCTs was deemed sufficient in 39.6% of the cases. There was no statistically significant difference in the quality of RCTs provided or the prevalence of procedural errors between maxillary and mandibular molars. RI resulted in more sufficient treatments compared with MI (49% vs. 30.3% respectively) (X^2^: 7.39, *p* = 0.007). The interrater reliability was excellent (Kappa statistic: 0.83).

Most students (93.1%) reported that they preferred RI over manual, while 6.9% of them had no preference. More than one quarter of the students (26.4%) reported that they did not feel confident to perform molar RCT without supervision.

Patients with procedural errors were informed of the unfortunate mishap and were either kept under observation, referred to a consultant endodontist or the post-graduate endodontic clinics, or, where the procedural errors were non-reparable, referred for extraction of the involved tooth.

## Discussion

This study demonstrated that procedural errors were very common, be it in manual or RI, and that students generally did not feel confident to perform molar RCT on their own without being supervised.

More than 60% of the treatments provided were judged to have either compromised the tooth structure, resulted in irreversible damage to the tooth or required further intervention before definitive restoration of the tooth could be completed. This is in agreement with the reported quality of RCT performed by UG students in different parts of the world [4–7]. RI only partially improved the quality of RCT and did not result in an acceptable level of sufficient treatments. A recent systematic review demonstrated a high tendency for procedural errors with MI [20].

The most common procedural error detected was under-extension of the root canal filling. The position of the apical constriction in relation to the radiographic apex is variable. In our study, 2 mm short of the radiographic apex was used as the cut-off point in accordance with previously published literature [6, 21, 22]. Under-extended root canal obturation could be explained by multiple reasons including errors in working length determination, ledge formation, lack of recapitulation during the step-back phase of instrumentation, separated instruments, improper master cone selection and improper obturation technique. The specific reason for this procedural error could not be identified in this observational study. Over-extension of the root canal filling was observed more often in MI. Lack of apical stop creation and overzealous instrumentation can be implicated for this procedural error.

Canal transportation was encountered more frequently following MI. It occurs due to the files’ inherent tendency to restore their original linear shape during canal instrumentation [23]. This is in accordance with other published studies [24, 25]. 

Obturation of the instrumented canals is essential and aims to entomb any residual infection and provide a fluid-tight seal against the ingress of fluids from the peri-radicular tissues [3]. Different techniques and materials have been described but none proved to be superior to the rest. In this study, obturation was completed using cold lateral condensation. Unexperienced clinicians may find this technique difficult to apply especially in narrow canals and in patients with limited mouth openings. This was evident in our results as almost half of the obturations had voids or were poorly condensed. The sealer-based obturation technique used with calcium silicate sealers can provide a simpler obturation option as it only requires the insertion of a single cone of gutta percha with no further condensation [26]. Coronal seal is an essential part of RCT [27]. Proper adaptation of a coronal restoration to sound tooth structure prevents the ingress of saliva into the obturated root canal system, and therefore prevents reinfection. A leaky coronal restoration (definitive or provisional) not only risks reinfection of the root canal system, but also exposes the tooth to recurrent caries which may compromise its restorability [27].

This study was based on radiographic assessment of the RCTs provided. While radiographs can reveal important mechanical aspects of RCT such as the extension, taper, and condensation of the root filling as well as the adaptation of the coronal restoration, they do not allow the assessment of the biological part of treatment [28]. Isolation during treatment, the irrigation protocol (solution(s), volume, time, activation… etc.), interappointment medication and quality and timing of coronal restoration are all factors that may influence the treatment outcome yet cannot be assessed radiographically. However, to achieve the best possible outcome, the mechanical steps of RCT should be executed to a very high standard and procedural errors should be avoided as much as possible. Poor technical quality of RCT was demonstrated to be a risk factor for apical periodontitis [9, 29]. 

The European society of endodontology’s undergraduate curriculum guidelines for endodontology recommend that “all students should gain adequate experience in the treatment of anterior, premolar and molar teeth in both the pre-clinical and clinical environment” [30]. They also state that clinical training should be based on competencies rather than a minimum number of performed procedures. Students should be trained to consider all treatment options, be competent at assessing tooth restorability and treatment complexity and to recognize when referral to a specialist should be considered [30]. 

This study has multiple limitations. It only evaluated the radiographic quality of molar RCTs. No clinical aspect was taken into consideration. Its lack of temporal factor precluded any observation of the success or failure of treatments and their association (or lack of) with the technical quality of the treatments provided. Clinical supervision varied between the participating students and there was no correlation between the students’ academic performance and the technical quality of RCTs. Potential confounding factors to the results include the level of academic performance of students, the variance in clinical supervision, the technical difficulty of the RCT provided, and the variation in outcome assessment between the assessors. No attempts to adjust for the confounding factors were made as the purpose of the study was to report on the prevalence of procedural errors rather than investigating the potential reasons for them. However, this study demonstrates an overall poor quality of molar RCT provided by UG students, combined with their lack of confidence to perform this procedure without supervision. This invites the question of whether UG students should be expected to perform molar RCT at such an early stage of their careers, or whether this procedure requires further training, mentorship and experience that may not be ideally delivered during undergraduate training. Inexperienced clinicians may benefit from a wider exposure to simple RCTs of anterior and premolar teeth before they can embark on the more technically demanding molar RCT. The authors suggest that UG endodontic clinical training should emphasize on providing simple RCTs on anterior and premolar teeth and on the conservative and emergency management of molar teeth such as vital pulp therapy, access cavity and coronal pulp extirpation, and incision and drainage. Clinicians who wish to perform molar RCT should receive further training following graduating from the dental school.

## Conclusion

The technical quality of molar RCT provided by UG students is generally insufficient. RI partially improved the quality of RCT compared with MI when used by inexperienced operators (UG students). Most students preferred RI over MI. UG students need further endodontic training and experience before they can safely and confidently practise molar RCT.

### Electronic supplementary material

Below is the link to the electronic supplementary material.

Supplementary Material 1

Supplementary Material 2

## Data Availability

The datasets used and/or analysed during the current study are available from the corresponding author on reasonable request.

## Abbreviations

MI: manual instrumentation
RCT: root canal treatment
RI: rotary instrumentation
UG: undergraduate
