# Supplementary material for: Molar root canal treatment performed by undergraduate dental students; an observational study of procedural errors and student perception
Source: BMC Med Educ. 2024 Apr 22;24:437. doi: 10.1186/s12909-024-05397-z (PMC11036572; doi:10.1186/s12909-024-05397-z)
Supplement: Supplementary file 1 — Supplementary Material 1 [file 12909_2024_5397_MOESM1_ESM.docx]

**Appendix 1: Definition of the investigated procedural errors:**

Under-extension of the root canal filling: extension of the root canal filling more than 2mm short of the radiographic apex.

Over-extension of the root canal filling: gutta percha extending beyond the radiographic apex (sealer puff is not included).

Improper apical instrumentation size: Apical preparation size is either too large or too small for that root.

Ledge: iatrogenic deviation from the canal pathway resulting in loss of the original canal pathway

Canal transportation: removal of canal wall structure on the outside curve in the apical half of the canal.

Missed canals: untreated canals detected on the post-obturation radiograph.

Access cavity-related perforation: a communication between the coronal root canal system and the external tooth surface that occurred during access cavity preparation either in the furcation area or the sides of the pulp chamber.

Instrumentation-related perforation: a communication between the radicular root canal system and the external tooth surface that occurred during canal instrumentation.

Strip perforation: a complete penetration of a root canal wall due to excessive lateral tooth structure removal during canal instrumentation.

Separated instrument: instrument separation within the root canal.

Sealer extrusion: extrusion of sealer into the periapical tissues without overextension of gutta percha.

Insufficient obturation: incomplete obturation of the root canal space with resultant voids

Improper access cavity: over or under-drilled access cavity.

Improper coronal seal: improper adaptation of the provisional or definitive coronal restoration which results in leakage of fluids from the oral cavity into the root canal system.

**Insufficient technical quality of RCT**: The presence of a procedural error or an irreversible damage to the tooth structure that can potentially compromise the outcome of RCT, or require further intervention before a satisfactory definitive restoration can be provided. Examples include, but not limited to, aggressive access cavity preparation, perforations, instrument separation, improper instrumentation and obturation, and improper coronal seal.
