# Supplementary material for: Molar root canal treatment performed by undergraduate dental students; an observational study of procedural errors and student perception
Source: BMC Med Educ. 2024 Apr 22;24:437. doi: 10.1186/s12909-024-05397-z (PMC11036572; doi:10.1186/s12909-024-05397-z)
Supplement: Supplementary file 2 — Supplementary Material 2 [file 12909_2024_5397_MOESM2_ESM.docx]

**Appendix 2: Data collected from the participating students:**

Student number: ___________________________________________________

**Case #1:**

Patient no.: _____________________________________________________

Tooth #: ________________________________________________________

Manual or rotary instrumentation: ___________________________________

Number of visits needed to complete the treatment: ____________________

**Case #2:**

Patient no.: _____________________________________________________

Tooth #: ________________________________________________________

Manual or rotary instrumentation: ___________________________________

Number of visits needed to complete the treatment: ____________________

Q1: Having completed the endodontic requirements, do you feel ready and confident to perform molar root canal treatment on your own without supervision? Yes / No

Q2: Having performed molar RCT using both the manual and rotary instrumentation techniques, which technique do you prefer?

*(Answer only if you completed 2 RCTs; one with manual and another with rotary instrumentation)*

Rotary / Manual / no preference
